# Main Inflammatory Cells and Potentials of Anti-Inflammatory Agents in Prostate Cancer

**DOI:** 10.3390/cancers11081153

**Published:** 2019-08-12

**Authors:** Takuji Hayashi, Kazutoshi Fujita, Makoto Matsushita, Norio Nonomura

**Affiliations:** Department of Urology, Osaka University Graduate School of Medicine, Suita, Osaka 565-0871, Japan

**Keywords:** inflammation, prostate cancer, immune cell, intervention, NSAIDs, metformin, statin, cytokine, mouse model

## Abstract

Prostate cancer is the most common type of cancer and the leading cause of cancer deaths among men in many countries. Preventing progression is a major concern for prostate cancer patients on active surveillance, patients with recurrence after radical therapies, and patients who acquired resistance to systemic therapies. Inflammation, which is induced by various factors such as infection, microbiome, obesity, and a high-fat diet, is the major etiology in the development of prostate cancer. Inflammatory cells play important roles in tumor progression. Various immune cells including tumor-associated neutrophils, tumor-infiltrating macrophages, myeloid-derived suppressor cells, and mast cells promote prostate cancer via various intercellular signaling. Further basic studies examining the relationship between the inflammatory process and prostate cancer progression are warranted. Interventions by medications and diets to control systemic and/or local inflammation might be effective therapies for prostate cancer progression. Epidemiological investigations and basic research using human immune cells or mouse models have revealed that non-steroidal anti-inflammatory drugs, metformin, statins, soy isoflavones, and other diets are potential interventions for preventing progression of prostate cancer by suppressing inflammation. It is essential to evaluate appropriate indications and doses of each drug and diet.

## 1. Introduction

Prostate cancer is the most common type of cancer and the leading cause of cancer deaths among men in many countries [1]. The patients with late stage disease, who exhibit poorly differentiated cancer cells, local invasion or metastatic lesion, have a poor prognosis. Although the patients with an early stage of the disease have a good prognosis after several treatments including radical prostatectomy, radiation therapy and hormonal therapy, these treatments have raised concerns about the various complications [2,3,4]. In order to avoid the complications, active surveillance has been one of the treatment options for early stage prostate cancer patients [5,6]. The prostate cancer patients with recurrence after radical prostatectomy and radiation therapy or patients who acquired resistance to systemic therapy have worried about disease progression. It is particularly important for these patients to suppress the progression of prostate cancer. Prostate cancer has a long natural history from the diagnosis to the death caused by cancer progression. The median survival was more than 12 years after diagnosis of localized prostate cancer at mean 67 years old [7], and more than 2 years even after diagnosis of metastatic prostate cancer at median 70 years old [8]. Thus, the progression of prostate cancer could be affected by the environmental factors, lifestyles, and chronic diseases.

Inflammation is the major etiology behind the development of prostate cancer. Acute or chronic inflammation can result in not only carcinogenesis but also progression of prostate cancer [9,10,11,12]. Inflammation in prostate cancer is linked to various factors including infection [13], microbiome [14], obesity [15], and high-fat diet (HFD) [16]. Inflammatory cells consist of innate immune cells and acquired immune cells. Innate immune cells are the main players in early phase of the inflammation and affect tumor progression via intercellular signaling including cytokines and chemokines [17].

Lifestyle, especially dietary habits, is the basis of chronic systemic inflammation, which also constitutes a risk for diabetes mellitus, cardiovascular disease, neurodegenerative diseases, and certain cancers including breast, colon, and pancreas cancer [18]. Chronic systemic inflammation results from the effects of dietary pattern and components on gut microbiota [19]. Dietary pattern can activate pro-inflammatory response in the prostate and modulate prostate cancer progression [20,21,22]. The gut microbiome could have indirect interactions with prostate cancers by altering the immune system. The urinary microbiome inducing chronic prostatic inflammation, and the presence of pro-inflammatory bacteria might cause prostate cancer progression [23]. Although it remains unclear how chronic systemic inflammation affects the local inflammation and cancer development in prostate, the relationship among dietary components, gut microbiome, and immune cells are thought to play major roles in the cascade.

It has been reported that the drugs and diets that suppress the inflammatory responses or modulating immune status have clinical benefits for prostate cancer patients (Table 1). Although some drugs show promise in epidemiological studies [24], any one drug is unlikely to be effective for preventing progression in all prostate cancer patients.

**Figure 1:**
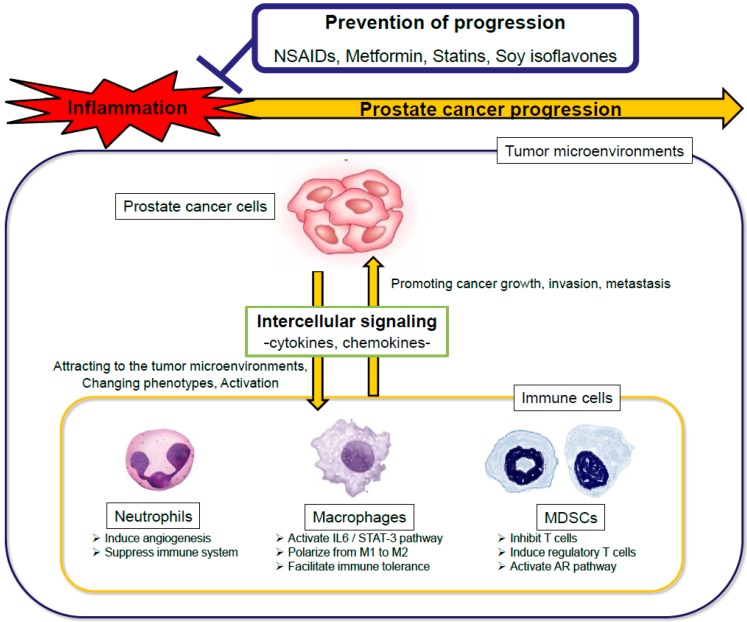
The scheme of the interactions of immune cells with prostate cancer progression and the interventions against inflammation. NSAIDs, non-steroidal anti-inflammatory drugs; IL, interleukin; STAT, signal transducer and activator of transcription; MDSCs, myeloid-derived suppressor cells; AR, androgen receptor.

In this review, we discuss the immune cells and intercellular signaling that promote prostate cancer progression. We also discuss recent findings pertaining to drugs and diets that may prevent prostate cancer progression by controlling the inflammation (Figure 1).

## 2. Immune Cells and Intercellular Signaling Promoting Prostate Cancer Progression

### 2.1. Neutrophils

Neutrophils are short-lived cells with a circulating half-life of less than 24 h [39] and primarily work as an antibacterial immune response. The cytokines secreted by tumor cells, such as granulocyte colony-stimulating factor (G-CSF), interleukin (IL)1β, IL6 or tumor necrosis factor (TNF), have been suggested to extend their lifespan [40,41]. Chemokines secreted from tumor cells also attract neutrophils in the blood to the tumor microenvironment, where they become tumor-associated neutrophils (TANs). TANs can be immunosuppressive, and stimulate tumor cell proliferation and angiogenesis (N2 TANs). However, they can also inhibit tumor growth (N1 TANs) [40]. TANs are also reported to play important roles on the metastatic cascade [41]. The administration of the tyrosine kinase inhibitor cabozantinib resulted in the clearance of invasive prostate cancer of *Pten*/*Tp53*-deficient (prostate-specific knockout) mouse model by recruiting neutrophils to the tumor [42].

Although murine neutrophils differ from human neutrophils in terms of surface markers and genetic diversity [43], evidence suggested that neutrophils play an important role in progression of human prostate cancers. The peripheral blood neutrophil-lymphocyte ratio is associated with a high Gleason score and a poor prognosis in early stage prostate cancer [44,45], and is also a prognostic factor for response to abiraterone and docetaxel treatment in late stage castration-resistant prostate cancer [46,47]. Other studies have shown that neutrophils in both peripheral blood and prostate are predictive factors for prostate biopsy results [48,49]. While correlative studies suggest a role for neutrophils in human prostate cancer, further research is needed to confirm their ability to drive disease progression.

### 2.2. Macrophages

Macrophages play important roles in promoting growth and bone metastasis of prostate cancer [50]. Monocyte chemotactic protein (MCP)-1/C-C motif ligand (CCL)2 secreted by prostate cancer cells and stromal cells recruits tumor-infiltrating macrophages and induces tumor progression [51,52]. Macrophages are one of the most abundant types of immune cells in the tumor microenvironments and divided into classic macrophages (M1) and alternative macrophages (M2). M1 macrophages act in microbiocidal and anti-tumor activity with the secretion of IL1β, IL12 and TNF-α, whereas M2 macrophages act in tissue remodeling, immune tolerance and tumor progression with the secretion of IL4, IL10 and transforming growth factor (TGF)-β [53,54]. Exposure of macrophages to IL4, CSF-1, granulocyte-macrophage colony-stimulating factor (GM-CSF) and TGF-β secreted by cancer cells polarize macrophages to the M2 phenotype, resulting in immunosuppressive microenvironments. Although obesity leads to a shift of the macrophage phenotype from M2 to M1 in the adipose tissues of mice [55], these macrophages also increased the expression of CD206, which is a surface marker of the M2 polarization phenotype [56]. In *Pten*-deficient model mice (<Pb-Cre+;*Pten*(fl/fl)>) of prostate cancer, the ratio of tumor-infiltrating macrophages expressing CD206 to ones expressing major histocompatibility complex (MHC) class II was increased, and IL6 secreted by tumor-infiltrating macrophages was elevated by HFD [25]. IL6 secreted by many cell types can promote cancer growth via phosphorylation of signal transducer and activator of transcription (STAT)-3 [57].

In humans, CD206-positive M2 tumor-infiltrating macrophages are associated with metastasis and poor prognosis [58], and were more abundant in the metastases of castration-resistant prostate cancer [59]. Tumor-infiltrating macrophages are partly derived from blood monocytes [60]. Peripheral blood monocyte fraction is increased in pathologically high-grade prostate cancer [61]. Peripheral high monocyte count, which reflects tumor-infiltrating macrophages [62], is a negative predictive factor for prostate cancer treated with hormonal therapy [63] and chemotherapy [64]. Circulating monocytes from prostate cancer patients may promote invasion of epithelial cells [65]. Detailed investigations of the roles of macrophages and monocytes in prostate cancer progression are needed.

### 2.3. Myeloid-Derived Suppressor Cells (MDSCs)

Myeloid-derived suppressor cells (MDSCs) are the immature myeloid cells that suppress anti-tumor immune responses in the tumor microenvironments. Inflammatory responses drive accumulation and activation of MDSCs [66]. MDSCs are a heterogeneous population and express a mixture of surface markers typical for myeloid cells, but lack the markers of lymphocytes, natural killer cells, macrophages and dendritic cells. MDSCs modulate the cytokine production of macrophages and promote tumor angiogenesis and metastasis [67]. MDSCs, which are characterized by the surface marker CD11b^+^Ly6C^+^Ly6G^+^ in mice, also inhibit T cells via arginase-1, inducible nitric oxide synthase (iNOS) and reactive oxygen species and induce regulatory T cells by IL10 and TGF-β. Reported inducers of MDSCs include lipopolysaccharide, CSF-1, GM-CSF, IL1β, IL6, IL13, and prostaglandin E2 (PGE2) [68]. In *Pten*-deficient model mice, MDSCs infiltration in tumor was increased and tumor growth was promoted by HFD [25]. C-X-C motif ligand (CXCL)5 secreted from prostate cancer cells attracts MDSCs expressing C-X-C chemokine receptor (CXCR)2, and the elimination of MDSCs or the blocking of CXCL5-CXCR2 signaling elicits antitumor responses in the *Pten*/*Smad4*-deficient mouse model [69]. In the transgenic adenocarcinoma of mouse prostate (TRAMP) model, IL23 secreted from MDSCs can activate the androgen receptor (AR) pathway, promoting cell survival and proliferation under androgen-deprived conditions, suggested a mechanism of MDSC-mediated resistance to castration [70]. MDSCs are divided into two major groups: The cells with a morphology and surface markers like monocytes (monocytic (M)-MDSCs, CD11b^+^Ly6C^high^Ly6G^−^) and the cells with those like neutrophils (polymorphonuclear (PMN)-MDSCs or granulocytic MDSCs, CD11b^+^Ly6C^low^ Ly6G^+^).

MDSCs were originally discovered in mice, and their counterparts in humans are not clearly defined. In humans, many studies report that the equivalent cells to PMN-MDSCs are defined as CD11b^+^CD14^−^CD15^+^ or CD11b^+^CD14^−^CD66b^+^, and that M-MDSCs are defined as CD11b^+^CD14^+^HLA-DR^−/low^ CD15^−^ [71]. Both PMN-MDSCs and M-MDSCs in peripheral blood from patients with prostate cancer were significantly increased compared with healthy donors, and were negatively associated with overall survival [72,73]. PMN-MDSCs in metastatic lymph nodes of prostate cancer exhibited a high degree of immunosuppressive activity [74]. Both peripheral blood and local MDSCs could be a new target in the prevention of prostate cancer progression.

### 2.4. Others

Mast cells have key roles in inflammation and allergy. Mast cells are known to release molecules to influence tumor growth [75]. Mast cells are heterogeneous population, and correlations between mast cell infiltration and prostate cancer prognosis have been controversial [76,77]. The numbers of tumor-infiltrating mast cells were not significantly different in *Pten*-deficient model mice between normal diet and HFD conditions [25]. Cross-talk between PMN-MDSCs and mast cells induce tumor-specific immunosuppression in TRAMP mice [78]. Further studies on the functions of mast cells in prostate cancer progression are warranted.

Other inflammatory and immune cells such as dendritic cells, natural killer (NK) cells, B cells and T cells could be also involved in the prostate cancer progression [79]. NK cells are a type of cytotoxic lymphocyte and have the ability of much faster immune response to tumor. B cells and T cells are the major cellular components of the adaptive immune response. B cells also play important roles in prostate cancer progression via intercellular signaling. Although T cells have anti-tumorigenic functions, some populations of T cells such as regulatory T cells have immunosuppressive functions. A hormonally induced mouse model for early stage prostate cancer progression exhibited early and persistent prostatic mast cell infiltration with subsequent accumulation of neutrophils, T cells, and macrophages, as well as increased expression of numerous chemokines [80].

There is growing recognition of the critical role of platelets in inflammation and cancer progression. Platelets release numerous inflammatory mediators and form bridges between leukocytes and endothelium by forming aggregates with leukocytes. Through their interactions with neutrophils, monocytes, lymphocytes and the endothelium, platelets are important coordinators of inflammation and both innate and adaptive immune responses. Moreover, platelets might be interacted with cancer metastasis by expressing P-selectin [81]. A meta-analysis showed that peripheral blood high platelet-lymphocyte ratio was correlated with poor prognosis in prostate cancer patients [82].

IL17 produced by T cells and other immune cells plays important roles in inflammation, autoimmune diseases, and prostate cancer. It was reported that IL17 signaling is required for the transition of prostatic intraepithelial neoplasia to adenocarcinoma and that IL17 promotes development of invasive prostate adenocarcinoma under castrate conditions by the experiments using IL17 receptor-knockout *Pten*-deficient model mice [83,84]. Liu S. et al. reported that hyperinsulinemia enhances IL17-induced inflammation to prostate cancer progression in obese mice [85].

Cyclooxygenase (COX)-2, an enzyme that catalyzes the rate-limiting step in prostaglandin and thromboxane production, is induced by various pro-inflammatory cytokines, and thought to promote prostate progression [86]. COX-2 inhibition could be effective for preventing progression of prostate cancer [87].

The activation of nuclear factor (NF)-κB, a major transcription factor that regulates inflammatory and immune responses, is associated with cancer progression. NF-κB is also a key mediator in metastasis and castration-resistance of prostate cancer [88].

## 3. Potential Effective Drugs and Diets for Preventing Progression of Prostate Cancer by Controlling the Inflammation 

### 3.1. Aspirin, Non-Steroidal Anti-Inflammatory Drugs (NSAIDs)

Observational study and randomized trials revealed that individuals taking aspirin and non-steroidal anti-inflammatory drugs (NSAIDs) had lower incidence of colon cancer [89,90,91]. Because inflammatory factors have been reported to induce initiation and progression of various types of cancer, NSAIDs are thought to not only reduce incidence but also prevent progression of cancer by suppressing various inflammatory pathways, including the COX-2 pathway [92]. In addition, NSAIDs inhibit cancer progression via inducing tumor cell apoptosis, protecting and repairing DNA damage, and suppressing the platelet activity [93].

In the REDUCE study, where all men had negative baseline biopsy, aspirin or NSAIDs use was associated with a reduced risk of total and high-grade prostate cancer [94]. The associations between aspirin or NSAIDs use and prostate cancer outcomes have also been analyzed in large studies [95,96,97,98,99]. In a study of localized prostate cancer treated with radical prostatectomy or radiation therapy, disease-specific mortality was significantly lower in aspirin users, the trend being driven prominently by high-risk patients [95]. There was no evidence of a protective association between pre-diagnosis use of low-dose aspirin and disease-specific mortality in the cohort of newly diagnosed prostate cancer [96]. Pre-diagnosed aspirin use was associated with a small reduced risk of disease-specific mortality (non-significant, hazard ratio = 0.88, 95% confidence interval 0.67–1.15), and higher dose aspirin users had stronger associations in one cohort [97]. In a subgroup analysis of one cohort, post-diagnosis aspirin use was significantly associated with lower disease-specific mortality in high-risk prostate cancer patients [98]. In the STAMPEDE trial, a randomized control trial of celecoxib (a selective COX-2 inhibitor) in addition to hormonal therapy in patients with locally advanced or metastatic prostate cancer, no benefit of celecoxib was observed [99]. A meta-analysis showed that prostate cancer patients exposed to pre- or post-diagnostic NSAIDs experienced a significantly reduced risk of distant metastasis [100], which may suggest the effects of NSAIDs on platelets.

It was reported that celecoxib, the dose of which was equivalent to the one clinically used in human, suppressed tumor growth, local MDSCs infiltration, M2 polarization of tumor-infiltrating macrophages, and IL6 secretion by tumor-infiltrating macrophages in *Pten*-deficient mouse model under HFD, but not under a normal diet [25]. In the model, mRNA expression of COX-2 (*Ptgs2*) were not altered by administration of HFD and celecoxib. These results suggested that celecoxib might have therapeutic benefits in the particular subgroup of prostate cancers such as obese patients, and that the local expression of COX-2 might not be a biomarker for the response to celecoxib in prostate cancer. Because we have no available data on obesity and local expression of COX-2 in the STAMPEDE trial, further investigations are needed.

Although NSAIDs could be effective in a subset of prostate cancer patients, the indications of these drugs have remained unclear. Mascan B. et al. reported that NSAIDs might have clinical benefits for prostate cancer patients undergoing radiation therapy as inflammation is a common side-effect of the therapy [101]. Biomarkers for the anti-tumor effects of NSAIDs may be the somatic *PIK3CA* mutation, the low levels of PD-L1, or the particular single-nucleotide polymorphism [102]. The US “Guidelines for the Use of Preventive Drugs” clearly state that daily intake equivalent to a “low-dose”, which means 75–100 mg per day of aspirin, has anticancer effects. However, taken into account the side effects of long-term use of aspirin such as gastrointestinal damage and exacerbate respiratory disease [102], making careful judgement is needed for long-term administration of aspirin or NSAIDs.

### 3.2. Metformin

Metformin, which is the most commonly used as oral anti-diabetic drug in the world, has been reported to lower cancer incidence and cancer-specific death [103,104]. Epidemiological studies demonstrated that metformin improves survival of prostate cancer patients [105,106,107,108,109,110,111,112], and may reduce incidence of prostate cancer [112,113,114].

Metformin is known to activate the enzyme adenosine mono-phosphate-activated protein kinase (AMPK) directly after energetic stress [115,116]. As a result of AMPK activation, mammalian target of rapamycin (mTOR) complex-1 (mTORC1) signaling is inhibited. Metformin also inhibits mTORC1 via an AMPK-independent pathway [117]. Metformin has been shown to inhibit the proliferation of prostate cancer [118,119]. Moreover, both the protein and mRNA expression of AR were reduced by metformin [120,121]. Metformin could be more effective in combinations with hormonal therapy or chemotherapy [118,122]. There have been many reports of the other indirect anticancer effects of metformin [123,124].

There are several mechanisms by which metformin inhibits the progression of prostate cancer are related with inflammation. Suppressing NF-κB pathway is a key event in activation of AMPK and inhibition of mTOR by metformin [26]. Tong D. et al. reported that metformin was capable of repressing epithelial-mesenchymal transition via reducing expression of COX-2/PGE2/phosphorylated STAT-3 [27]. In the TRAMP mouse model, metformin delays prostate cancer progression with concurrent reductions in recruitment of macrophages and downregulation of both COX-2 and PGE2 in tumor cells [28]. Metformin reduces prostate cancer growth prominently under HFD by modulating multiple signaling pathways in xenograft mice [125]. In *Pten*-deficient model mice, metformin also inhibits prostate cancer growth with reducing local MDSCs under HFD, but not under a normal diet [29]. These findings suggest that metformin could have clinical benefits for prostate cancer partly by suppressing inflammatory infiltration. In the various other cancer mouse models, metformin is reported to have an anti-tumor effect on macrophages [30,31], MDSCs [31], and CD8^+^ T cells [32].

Further studies are necessary to determine which dose of metformin could be clinically beneficial and safely administered to prostate cancer patients, and if metformin would be effective in non-diabetic populations [123,126].

### 3.3. Statins

Statins are widely used for the treatment of lipid disorders. Preclinical and clinical evidence suggest that statins have anti-tumor activity in various types of cancer [127,128]. Some epidemiological studies showed that statin use was not associated with reduced risk of total or advanced prostate cancer [129,130,131,132]. However, other studies found that it was associated with a reduction in prostate cancer risk, particularly advanced disease risk [133,134,135,136,137]. Prostate cancer patients who used statins had significantly lower disease-specific mortality than nonusers [138,139,140,141], whereas statin use was not associated with biochemical recurrence among the patients treated with radical prostatectomy [140,141]. One case-control study demonstrated that the effect on prostate cancer mortality was dependent on statin type (strong: Cerivastatin, atorvastatin, and simvastatin/weak: Pravastatin, lovastatin, and fluvastatin) used [142]. Pon D et al. suggested that the benefit of statins may be more pronounced in the prostate cancer patients who take statins for a prolonged period of time (>12 months) [143].

Statins are reported to suppress prostate cancer progression by inhibiting inflammation, angiogenesis, cell proliferation, migration, adhesion, and invasion, and by promoting apoptosis [144,145]. Dysregulation of cholesterol homeostasis in prostate tumors induces elevation of intracellular cholesterol. As a result of the intracellular elevation, specialized cholesterol-rich regions of the cell membrane known as lipid rafts [146] facilitate cell signaling pathways involving the AR [147] and the epidermal growth factor receptor [148]. Statins are thought to disrupt the organization of the lipid rafts and suppress these intracellular signaling pathways.

It was reported that statins could have effects to reduce local inflammation by several studies using histological examination. Preoperative statin use was significantly associated with lower incidence of inflammation within prostate tumors of men undergoing radical prostatectomy [149]. Statin users have reduced prostate inflammation, relative to non-users in men with a negative prostate biopsy [150]. Murtola T.J. et al. reported the results of a randomized clinical trial using atorvastatin for a median of 27 days before radical prostatectomy [151]. Although prostate inflammation did not differ between the atorvastatin group and the placebo group, Ki-67 index was lowered in the atorvastatin arm in a time-dependent manner.

Several mechanisms by which statins affect inflammation have been reported. Cholesterol crystals in the blood induce production of pro-inflammatory cytokines (IL1β and IL6), resulting in the production of C-reactive protein (CRP) [152]. Statins disrupt this process by lowering cholesterol. Statins also reduce CRP level in a cholesterol-independent manner [153]. Moreover, statins could reduce the synthesis of MCP-1 [33] and may be associated with decreased level of CD11b adhesion molecule [34], which is a surface marker of macrophages and MDSCs. Statins are reported to increase the number of CD4^+^CD25^+^ regulatory T cells, which prevent various immunoinflammatory diseases by suppressing immune responses [35], by inducing the transcription factor, forkhead box P3 [154]. Statins can also reduce inducible MHC class II expression in antigen presenting cells, thereby inhibiting T cell activation [36], and activate peroxisome proliferator-activated receptors (PPARs), inhibiting inflammation [37].

Further investigations are needed to determine if statins should be administered to prostate cancer patients without assessment of their cholesterol profile and which types of statins have the most clinical benefits in prostate cancer [145].

### 3.4. Others

Soybeans are rich sources of bioactive phytochemicals, isoflavones. A meta-analysis of eight randomized control trials supported a possible role for soy and isoflavones in prostate cancer risk reduction [155]. Soy bread consumption for 56 days reduced MDSC-associated cytokines (IL6, GM-CSF, G-CSF, and CSF-1) and M-MDSCs in peripheral blood of prostate cancer patients with asymptomatic biochemical recurrence [38]. Although a randomized control trial failed to find a significant benefit to soy protein supplementation on the biochemical recurrence of prostate cancer after radical prostatectomy [156], soy isoflavones-rich diets may suppress inflammation and could prevent progression of prostate cancer.

Some other nutrients or foods including vitamin D, pomegranate, green tea, resveratrol, and zyflamend might be effective for preventing progression of prostate cancer by inhibiting inflammation [157,158].

## 4. Conclusions

Inflammation enhanced by various immune cells and intercellular signaling is one of important factors for progression of prostate cancer. There is a great deal of evidence suggesting the progression can be prevented by targeting inflammation. Further analysis in mouse models would give new insights into the mechanisms of the prostate cancer progression induced by inflammation and identify new therapeutic targets. Moreover, clinical trials would suggest the appropriate indications and doses of each drug or diet to prevent progression of prostate cancer.

## Figures and Tables

**Table 1:** Potential drugs and diets for preventing prostate cancer progression by controlling the inflammation.

Drugs or Diets	Mechanism of Action
Aspirin, NSAIDs	Inhibit COX-2 pathway
	Suppress local MDSC infiltration [25]
	Prevent M2 polarization of tumor-infiltrating macrophages [25]
	Reduce IL6 secretion by tumor-infiltrating macrophages [25]
Metformin	Suppress NF-κB pathway [26]
	Downregulate COX-2 and PGE2 in tumor cells [27,28]
	Reduce tumor-infiltrating macrophages [28]
	Inhibit local MDSC infiltration [29]
	Prevent M2 polarization of tumor-infiltrating macrophages [30]
	Promote M1 polarization of tumor-infiltrating macrophages [31]
	Reduce MDSCs in spleen and tumor [31]
	Protect exhaustion of CD8^+^ T cells in tumor [32]
Statins	Disrupt the organization of the lipid rafts
	Prevent the organization of cholesterol crystals
	Reduce the synthesis of MCP-1 [33]
	Decrease level of CD11b adhesion molecule [34]
	Increase regulatory T cells [35]
	Inhibit T cell activation [36]
	Activate peroxisome proliferator-activated receptors [37]
Soy isoflavones	Reduce MDSC-associated cytokines in peripheral blood [38]
	Reduce MDSCs in peripheral blood [38]
Vitamin D,	Unclear
Pomegranate,	
Green Tea,	
Resveratrol,	
Zyflamend	

Abbreviation: NSAIDs, non-steroidal anti-inflammatory drugs; COX, cyclooxygenase; MDSC, myeloid-derived suppressor cell; IL, interleukin; NF, nuclear factor; PGE2, prostaglandin E2; MCP, monocyte chemotactic protein.

## References

[1] Fitzmaurice C., Akinyemiju T.F., Al Lami F.H., Alam T., Alizadeh-Navaei R., Allen C., Alsharif U., Alvis-Guzman N., Amini E., Anderson B.O. (2018). Global, Regional, and National Cancer Incidence, Mortality, Years of Life Lost, Years Lived With Disability, and Disability-Adjusted Life-Years for 29 Cancer Groups, 1990 to 2016: A Systematic Analysis for the Global Burden of Disease Study. JAMA Oncol..

[2] De Carlo F., Celestino F., Verri C., Masedu F., Liberati E., Di Stasi S.M. (2014). Retropubic, laparoscopic, and robot-assisted radical prostatectomy: Surgical, oncological, and functional outcomes: A systematic review. Urol. Int..

[3] Amin N.P., Sher D.J., Konski A.A. (2014). Systematic review of the cost effectiveness of radiation therapy for prostate cancer from 2003 to 2013. Appl. Health Econ. Health Policy.

[4] Mitsuzuka K., Arai Y. (2018). Metabolic changes in patients with prostate cancer during androgen deprivation therapy. Int. J. Urol..

[5] Amin M.B., Lin D.W., Gore J.L., Srigley J.R., Samaratunga H., Egevad L., Rubin M., Nacey J., Carter H.B., Klotz L. (2014). The critical role of the pathologist in determining eligibility for active surveillance as a management option in patients with prostate cancer: Consensus statement with recommendations supported by the College of American Pathologists, International Society of Urological Pathology, Association of Directors of Anatomic and Surgical Pathology, the New Zealand Society of Pathologists, and the Prostate Cancer Foundation. Arch. Pathol. Lab. Med..

[6] Klotz L. (2019). Contemporary approach to active surveillance for favorable risk prostate cancer. Asian J. Urol..

[7] Wilt T.J., Jones K.M., Barry M.J., Andriole G.L., Culkin D., Wheeler T., Aronson W.J., Brawer M.K. (2017). Follow-up of Prostatectomy versus Observation for Early Prostate Cancer. N. Engl. J. Med..

[8] Bandini M., Pompe R.S., Marchioni M., Zaffuto E., Gandaglia G., Fossati N., Cindolo L., Montorsi F., Briganti A., Saad F. (2018). Improved cancer-specific free survival and overall free survival in contemporary metastatic prostate cancer patients: A population-based study. Int. Urol. Nephrol..

[9] De Marzo A.M., Platz E.A., Sutcliffe S., Xu J., Grönberg H., Drake C.G., Nakai Y., Isaacs W.B., Nelson W.G. (2007). Inflammation in prostate carcinogenesis. Nat. Rev. Cancer.

[10] Nakai Y., Nonomura N. (2013). Inflammation and prostate carcinogenesis. Int. J. Urol..

[11] Taverna G., Pedretti E., Di Caro G., Borroni E.M., Marchesi F., Grizzi F. (2015). Inflammation and prostate cancer: Friends or foe?. Inflamm. Res..

[12] Schillaci O., Scimeca M., Trivigno D., Chiaravalloti A., Facchetti S., Anemona L., Bonfiglio R., Santeusanio G., Tancredi V., Bonanno E. (2019). Prostate cancer and inflammation: A new molecular imaging challenge in the era of personalized medicine. Nucl. Med. Biol..

[13] Koul H.K., Kumar B., Koul S., Deb A.A., Hwa J.S., Maroni P., van Bokhoven A., Lucia M.S., Kim F.J., Meacham R.B. (2010). The role of inflammation and infection in prostate cancer: Importance in prevention, diagnosis and treatment. Drugs Today (Barc.).

[14] Sfanos K.S., Yegnasubramanian S., Nelson W.G., De Marzo A.M. (2018). The inflammatory microenvironment and microbiome in prostate cancer development. Nat. Rev. Urol..

[15] Fujita K., Hayashi T., Matsushita M., Uemura M., Nonomura N. (2019). Obesity, Inflammation, and Prostate Cancer. J. Clin. Med..

[16] Narita S., Nara T., Sato H., Koizumi A., Huang M., Inoue T., Habuchi T. (2019). Research Evidence on High-Fat Diet-Induced Prostate Cancer Development and Progression. J. Clin. Med..

[17] Shalapour S., Karin M., Shalapour S., Karin M. (2015). Immunity, inflammation, and cancer: An eternal fight between good and evil. J. Clin. Invest..

[18] Ruiz-Núñez B., Pruimboom L., Dijck-Brouwer D.A., Muskiet F.A. (2013). Lifestyle and nutritional imbalances associated with Western diseases: Causes and consequences of chronic systemic low-grade inflammation in an evolutionary context. J. Nutr. Biochem..

[19] Telle-Hansen V.H., Holven K.B., Ulven S.M. (2018). Impact of a Healthy Dietary Pattern on Gut Microbiota and Systemic Inflammation in Humans. Nutrients.

[20] Shankar E., Vykhovanets E.V., Vykhovanets O.V., Maclennan G.T., Singh R., Bhaskaran N., Shukla S., Gupta S. (2012). High-fat diet activates pro-inflammatory response in the prostate through association of Stat-3 and NF-κB. Prostate.

[21] Kobayashi N., Barnard R.J., Said J., Hong-Gonzalez J., Corman D.M., Ku M., Doan N.B., Gui D., Elashoff D., Cohen P. (2008). Effect of low-fat diet on development of prostate cancer and Akt phosphorylation in the Hi-Myc transgenic mouse model. Cancer Res..

[22] Blando J., Moore T., Hursting S., Jiang G., Saha A., Beltran L., Shen J., Repass J., Strom S., DiGiovanni J. (2011). Dietary energy balance modulates prostate cancer progression in Hi-Myc mice. Cancer Prev. Res. (Phila.).

[23] Porter C.M., Shrestha E., Peiffer L.B., Sfanos K.S. (2018). The microbiome in prostate inflammation and prostate cancer. Prostate Cancer Prostatic Dis..

[24] Campi R., Brookman-May S.D., Subiela Henríquez J.D., Akdoğan B., Brausi M., Klatte T., Langenhuijsen J.F., Linares-Espinos E., Marszalek M., Roupret M. (2018). Impact of Metabolic Diseases, Drugs, and Dietary Factors on Prostate Cancer Risk, Recurrence, and Survival: A Systematic Review by the European Association of Urology Section of Oncological Urology. Eur. Urol. Focus.

[25] Hayashi T., Fujita K., Nojima S., Hayashi Y., Nakano K., Ishizuya Y., Wang C., Yamamoto Y., Kinouchi T., Matsuzaki K. (2018). High-Fat Diet-Induced Inflammation Accelerates Prostate Cancer Growth via IL6 Signaling. Clin. Cancer Res..

[26] Kim H.G., Hien T.T., Han E.H., Hwang Y.P., Choi J.H., Kang K.W., Kwon K.I., Kim B.H., Kim S.K., Song G.Y. (2011). Metformin inhibits P-glycoprotein expression via the NF-κB pathway and CRE transcriptional activity through AMPK activation. Br. J. Pharmacol..

[27] Tong D., Liu Q., Liu G., Xu J., Lan W., Jiang Y., Xiao H., Zhang D., Jiang J. (2017). Metformin inhibits castration-induced EMT in prostate cancer by repressing COX2/PGE2/STAT3 axis. Cancer Lett..

[28] Liu Q., Tong D., Liu G., Gao J., Wang L.A., Xu J., Yang X., Xie Q., Huang Y., Pang J. (2018). Metformin Inhibits Prostate Cancer Progression by Targeting Tumor-Associated Inflammatory Infiltration. Clin. Cancer Res..

[29] Hayashi T., Fujita K., Matsushita M., Hayashi Y., Uemura M., Nonomura N. (2019). Metformin inhibits prostate cancer growth induced by a high-fat diet in *Pten*-deficient model mice. Int. J. Urol..

[30] Incio J., Tam J., Rahbari N.N., Suboj P., McManus D.T., Chin S.M., Vardam T.D., Batista A., Babykutty S., Jung K. (2016). PlGF/VEGFR-1 signaling promotes macrophage polarization and accelerated tumor progression in obesity. Clin. Cancer Res..

[31] Uehara T., Eikawa S., Nishida M., Kunisada Y., Yoshida A., Fujiwara T., Kunisada T., Ozaki T., Udono H. (2019). Metformin induces CD11b+-cell-mediated growth inhibition of an osteosarcoma: Implications for metabolic reprogramming of myeloid cells and anti-tumor effects. Int. Immunol..

[32] Eikawa S., Nishida M., Mizukami S., Yamazaki C., Nakayama E., Udono H. (2015). Immune-mediated antitumor effect by type 2 diabetes drug, metformin. Proc. Natl. Acad. Sci. USA.

[33] Romano M., Diomede L., Sironi M., Massimiliano L., Sottocorno M., Polentarutti N., Guglielmotti A., Albani D., Bruno A., Fruscella P. (2000). Inhibition of monocyte chemotactic protein-1 synthesis by statins. Lab. Invest..

[34] Weber C., Erl W., Weber K.S., Weber P.C. (1997). HMG-CoA reductase inhibitors decrease CD11b expression and CD11b-dependent adhesion of monocytes to endothelium and reduce increased adhesiveness of monocytes isolated from patients with hypercholesterolemia. J. Am. Coll. Cardiol..

[35] Mallat Z., Ait-Oufella H., Tedgui A. (2005). Regulatory T cell responses: Potential role in the control of atherosclerosis. Curr. Opin. Lipidol..

[36] Kwak B., Mulhaupt F., Myit S., Mach F. (2000). Statins as a newly recognized type of immunomodulator. Nat. Med..

[37] Paumelle R., Staels B. (2007). Peroxisome proliferator-activated receptors mediate pleiotropic actions of statins. Circ. Res..

[38] Lesinski G.B., Reville P.K., Mace T.A., Young G.S., Ahn-Jarvis J., Thomas-Ahner J., Vodovotz Y., Ameen Z., Grainger E., Riedl K. (2015). Consumption of soy isoflavone enriched bread in men with prostate cancer is associated with reduced proinflammatory cytokines and immunosuppressive cells. Cancer Prev. Res. (Phila.).

[39] Lahoz-Beneytez J., Elemans M., Zhang Y., Ahmed R., Salam A., Block M., Niederalt C., Asquith B., Macallan D. (2016). Human neutrophil kinetics: Modeling of stable isotope labeling data supports short blood neutrophil half-lives. Blood.

[40] Shaul M.E., Fridlender Z.G. (2019). Tumour-associated neutrophils in patients with cancer. Nat. Rev. Clin. Oncol..

[41] Coffelt S.B., Wellenstein M.D., de Visser K.E. (2016). Neutrophils in cancer: Neutral no more. Nat. Rev. Cancer.

[42] Patnaik A., Swanson K.D., Csizmadia E., Solanki A., Landon-Brace N., Gehring M.P., Helenius K., Olson B.M., Pyzer A.R., Wang L.C. (2017). Cabozantinib Eradicates Advanced Murine Prostate Cancer by Activating Antitumor Innate Immunity. Cancer Discov..

[43] Eruslanov E.B., Singhal S., Albelda S.M. (2017). Mouse versus Human Neutrophils in Cancer: A Major Knowledge Gap. Trends Cancer.

[44] Özsoy M., Moschini M., Fajkovic H., Soria F., Seitz C., Klatte T., Gust K., Briganti A., Karakiewicz P.I., Roupret M. (2018). Elevated preoperative neutrophil–lymphocyte ratio predicts upgrading at radical prostatectomy. Prostate Cancer Prostatic Dis..

[45] Jang W.S., Cho K.S., Kim M.S., Yoon C.Y., Kang D.H., Kang Y.J., Jeong W.S., Ham W.S., Choi Y.D. (2017). The prognostic significance of postoperative neutrophil-to-lymphocyte ratio after radical prostatectomy for localized prostate cancer. Oncotarget.

[46] Boegemann M., Schlack K., Thomes S., Steinestel J., Rahbar K., Semjonow A., Schrader A., Aringer M., Krabbe L.-M. (2017). The Role of the Neutrophil to Lymphocyte Ratio for Survival Outcomes in Patients with Metastatic Castration-Resistant Prostate Cancer Treated with Abiraterone. Int. J. Mol. Sci..

[47] Fan L., Wang R., Chi C., Cai W., Zhang Y., Qian H., Shao X., Wang Y., Xu F., Pan J. (2018). Systemic immune-inflammation index predicts the combined clinical outcome after sequential therapy with abiraterone and docetaxel for metastatic castration-resistant prostate cancer patients. Prostate.

[48] Fujita K., Imamura R., Tanigawa G., Nakagawa M., Hayashi T., Kishimoto N., Hosomi M., Yamaguchi S. (2012). Low serum neutrophil count predicts a positive prostate biopsy. Prostate Cancer Prostatic Dis..

[49] Fujita K., Hosomi M., Tanigawa G., Okumi M., Fushimi H., Yamaguchi S. (2011). Prostatic inflammation detected in initial biopsy specimens and urinary pyuria are predictors of negative repeat prostate biopsy. J. Urol..

[50] Lo C.H., Lynch C.C. (2018). Multifaceted Roles for Macrophages in Prostate Cancer Skeletal Metastasis. Front. Endocrinol. (Lausanne).

[51] Zhang J., Lu Y., Pienta K.J. (2010). Multiple roles of chemokine (C-C motif) ligand 2 in promoting prostate cancer growth. J. Natl. Cancer Inst..

[52] Fujita K., Ewing C.M., Getzenberg R.H., Parsons J.K., Isaacs W.B., Pavlovich C.P. (2010). Monocyte chemotactic protein-1 (MCP-1/CCL2) is associated with prostatic growth dysregulation and benign prostatic hyperplasia. Prostate.

[53] Wang J., Li D., Cang H., Guo B. (2019). Crosstalk between cancer and immune cells: Role of tumor-associated macrophages in the tumor microenvironment. Cancer Med..

[54] Corrêa L.H., Corrêa R., Farinasso C.M., de Sant’Ana Dourado L.P., Magalhães K.G. (2017). Adipocytes and Macrophages Interplay in the Orchestration of Tumor Microenvironment: New Implications in Cancer Progression. Front. Immunol..

[55] Mclaughlin T., Shen L., Engleman E., Mclaughlin T., Ackerman S.E., Shen L., Engleman E. (2017). Role of innate and adaptive immunity in obesity-associated metabolic disease. J. Clin. Invest..

[56] Haase J., Weyer U., Immig K., Klöting N., Blüher M., Eilers J., Bechmann I., Gericke M. (2014). Local proliferation of macrophages in adipose tissue during obesity-induced inflammation. Diabetologia.

[57] Rossi J.F., Lu Z.Y., Jourdan M., Klein B. (2015). Interleukin-6 as a therapeutic target. Clin. Cancer Res..

[58] Hu W., Qian Y., Yu F., Liu W., Wu Y., Fang X., Hao W. (2015). Alternatively activated macrophages are associated with metastasis and poor prognosis in prostate adenocarcinoma. Oncol. Lett..

[59] Zarif J.C., Baena-Del Valle J.A., Hicks J.L., Heaphy C.M., Vidal I., Luo J., Lotan T.L., Hooper J.E., Isaacs W.B., Pienta K.J. (2019). Mannose Receptor-positive Macrophage Infiltration Correlates with Prostate Cancer Onset and Metastatic Castration-resistant Disease. Eur. Urol. Oncol..

[60] Elliott L.A., Doherty G.A., Sheahan K., Ryan E.J. (2017). Human Tumor-Infiltrating Myeloid Cells: Phenotypic and Functional Diversity. Front. Immunol..

[61] Hayashi T., Fujita K., Tanigawa G., Kawashima A., Nagahara A., Ujike T., Uemura M., Takao T., Yamaguchi S., Nonomura N. (2017). Serum monocyte fraction of white blood cells is increased in patients with high Gleason score prostate cancer. Oncotarget.

[62] Hayashi T., Fujita K., Nojima S., Hayashi Y., Nakano K., Ishizuya Y., Wang C., Yamamoto Y., Kinouchi T., Matsuzaki K. (2017). Peripheral blood monocyte count reflecting tumor-infiltrating macrophages is a predictive factor of adverse pathology in radical prostatectomy specimens. Prostate.

[63] Wang Y.Q., Zhu Y.J., Pan J.H., Xu F., Shao X.G., Sha J.J., Liu Q., Huang Y.R., Dong B.J., Xue W. (2017). Peripheral monocyte count: An independent diagnostic and prognostic biomarker for prostate cancer—A large Chinese cohort study. Asian J. Androl..

[64] Shigeta K., Kosaka T., Kitano S., Yasumizu Y., Miyazaki Y., Mizuno R., Shinojima T., Kikuchi E., Miyajima A., Tanoguchi H. (2016). High Absolute Monocyte Count Predicts Poor Clinical Outcome in Patients with Castration-Resistant Prostate Cancer Treated with Docetaxel Chemotherapy. Ann. Surg. Oncol..

[65] Cavassani K.A., Meza R.J., Habiel D.M., Chen J.F., Montes A., Tripathi M., Martins G.A., Crother T.R., You S., Hogaboam C.M. (2018). Circulating monocytes from prostate cancer patients promote invasion and motility of epithelial cells. Cancer Med..

[66] Ostrand-Rosenberg S., Sinha P. (2009). Myeloid-derived suppressor cells: Linking inflammation and cancer. J. Immunol..

[67] Millrud C.R., Bergenfelz C., Leandersson K. (2017). On the origin of myeloid-derived suppressor cells. Oncotarget.

[68] Zhao Y., Wu T., Shao S., Shi B., Zhao Y. (2015). Phenotype, development, and biological function of myeloid-derived suppressor cells. Oncoimmunology.

[69] Wang G., Lu X., Dey P., Deng P., Wu C.C., Jiang S., Fang Z., Zhao K., Konaparthi R., Hua S. (2016). Targeting YAP-Dependent MDSC Infiltration Impairs Tumor Progression. Cancer Discov..

[70] Calcinotto A., Spataro C., Zagato E., Di Mitri D., Gil V., Crespo M., De Bernardis G., Losa M., Mirenda M., Pasquini E. (2018). IL-23 secreted by myeloid cells drives castration-resistant prostate cancer. Nature.

[71] Bronte V., Brandau S., Chen S.H., Colombo M.P., Frey A.B., Greten T.F., Mandruzzato S., Murray P.J., Ochoa A., Ostrand-Rosenberg S. (2016). Recommendations for myeloid-derived suppressor cell nomenclature and characterization standards. Nat. Commun..

[72] Chi N., Tan Z., Ma K., Bao L., Yun Z. (2014). Increased circulating myeloid-derived suppressor cells correlate with cancer stages, interleukin-8 and -6 in prostate cancer. Int. J. Clin. Exp. Med..

[73] Idorn M., Køllgaard T., Kongsted P., Sengeløv L., thor Straten P. (2014). Correlation between frequencies of blood monocytic myeloid-derived suppressor cells, regulatory T cells and negative prognostic markers in patients with castration-resistant metastatic prostate cancer. Cancer Immunol. Immunother..

[74] Sharma V., Dong H., Kwon E., Karnes R.J. (2018). Positive Pelvic Lymph Nodes in Prostate Cancer Harbor Immune Suppressor Cells To Impair Tumor-reactive T Cells. Eur. Urol. Focus..

[75] Pittoni P., Colombo M.P. (2012). The dark side of mast cell-targeted therapy in prostate cancer. Cancer Res..

[76] Nonomura N., Takayama H., Nishimura K., Oka D., Nakai Y., Shiba M., Tsujimura A., Nakayama M., Aozasa K., Okuyama A. (2007). Decreased number of mast cells infiltrating into needle biopsy specimens leads to a better prognosis of prostate cancer. Br. J. Cancer.

[77] Fleischmann A., Schlomm T., Köllermann J., Sekulic N., Huland H., Mirlacher M., Sauter G., Simon R., Erbersdobler A. (2009). Immunological microenvironment in prostate cancer: High mast cell densities are associated with favorable tumor characteristics and good prognosis. Prostate.

[78] Jachetti E., Cancila V., Rigoni A., Bongiovanni L., Cappetti B., Belmonte B., Enriquez C., Casalini P., Ostano P., Frossi B. (2018). Cross-Talk between Myeloid-Derived Suppressor Cells and Mast Cells Mediates Tumor-Specific Immunosuppression in Prostate Cancer. Cancer Immunol. Res..

[79] Strasner A., Karin M. (2015). Immune Infiltration and Prostate Cancer. Front. Oncol..

[80] Ellem S.J., Wang H., Poutanen M., Risbridger G.P. (2009). Increased endogenous estrogen synthesis leads to the sequential induction of prostatic inflammation (prostatitis) and prostatic pre-malignancy. Am. J. Pathol..

[81] Thomas M.R., Storey R.F. (2015). The role of platelets in inflammation. Thromb. Haemost..

[82] Wang J., Zhou X., He Y., Chen X., Liu N., Ding Z., Li J. (2018). Prognostic role of platelet to lymphocyte ratio in prostate cancer: A meta-analysis. Medicine (Baltimore).

[83] Zhang Q., Liu S., Ge D., Zhang Q., Xue Y., Xiong Z., Abdel-Mageed A.B., Myers L., Hill S.M., Rowan B.G. (2012). Interleukin-17 promotes formation and growth of prostate adenocarcinoma in mouse models. Cancer Res..

[84] Zhang Q., Liu S., Zhang Q., Xiong Z., Wang A.R., Myers L., Melamed J., Tang W.W., You Z. (2014). Interleukin-17 promotes development of castration-resistant prostate cancer potentially through creating an immunotolerant and pro-angiogenic tumor microenvironment. Prostate.

[85] Liu S., Zhang Q., Chen C., Ge D., Qu Y., Chen R., Fan Y.M., Li N., Tang W.W., Zhang W. (2016). Hyperinsulinemia enhances interleukin-17-induced inflammation to promote prostate cancer development in obese mice through inhibiting glycogen synthase kinase 3-mediated phosphorylation and degradation of interleukin-17 receptor. Oncotarget.

[86] Stock D., Groome P.A., Siemens D.R. (2008). Inflammation and prostate cancer: A future target for prevention and therapy?. Urol. Clin. North. Am..

[87] Sooriakumaran P., Langley S.E., Laing R.W., Coley H.M. (2007). COX-2 inhibition: A possible role in the management of prostate cancer?. J. Chemother..

[88] Nguyen D.P., Li J., Yadav S.S., Tewari A.K. (2014). Recent insights into NF-κB signalling pathways and the link between inflammation and prostate cancer. BJU Int..

[89] Kune G.A., Kune S., Watson L.F. (1988). Colorectal cancer risk, chronic illnesses, operations, and medications: Case control results from the Melbourne Colorectal Cancer Study. Cancer Res..

[90] Baron J.A., Cole B.F., Sandler R.S., Haile R.W., Ahnen D., Bresalier R., McKeown-Eyssen G., Summers R.W., Rothstein R., Burke C.A. (2003). A randomized trial of aspirin to prevent colorectal adenomas. N. Engl. J. Med..

[91] Benamouzig R., Deyra J., Martin A., Girard B., Jullian E., Piednoir B., Couturier D., Coste T., Little J., Chaussade S. (2003). Daily soluble aspirin and prevention of colorectal adenoma recurrence: One-year results of the APACC trial. Gastroenterology.

[92] Kashfi K. (2009). Anti-inflammatory agents as cancer therapeutics. Adv. Pharmacol..

[93] Zhang Z., Chen F., Shang L. (2018). Advances in antitumor effects of NSAIDs. Cancer Manag. Res..

[94] Vidal A.C., Howard L.E., Moreira D.M., Castro-Santamaria R., Andriole G.L., Freedland S.J. (2015). Aspirin, NSAIDs, and risk of prostate cancer: Results from the REDUCE study. Clin. Cancer Res..

[95] Choe K.S., Cowan J.E., Chan J.M., Carroll P.R., D’Amico A.V., Liauw S.L. (2012). Aspirin use and the risk of prostate cancer mortality in men treated with prostatectomy or radiotherapy. J. Clin. Oncol..

[96] Cardwell C.R., Flahavan E.M., Hughes C.M., Coleman H.G., O’Sullivan J.M., Powe D.G., Murray L.J. (2014). Low-dose aspirin and survival in men with prostate cancer: A study using the UK Clinical Practice Research Datalink. Cancer Causes Control.

[97] Flahavan E.M., Bennett K., Sharp L., Barron T.I. (2014). A cohort study investigating aspirin use and survival in men with prostate cancer. Ann. Oncol..

[98] Jacobs E.J., Newton C.C., Stevens V.L., Campbell P.T., Freedland S.J., Gapstur S.M. (2014). Daily aspirin use and prostate cancer-specific mortality in a large cohort of men with nonmetastatic prostate cancer. J. Clin. Oncol..

[99] James N.D., Sydes M.R., Mason M.D., Clarke N.W., Anderson J., Dearnaley D.P., Dwyer J., Jovic G., Ritchie A.W., Russell J. (2012). Celecoxib plus hormone therapy versus hormone therapy alone for hormone-sensitive prostate cancer: First results from the STAMPEDE multiarm, multistage, randomised controlled trial. Lancet Oncol..

[100] Zhao X., Xu Z., Li H. (2017). NSAIDs Use and Reduced Metastasis in Cancer Patients: Results from a meta-analysis. Sci. Rep..

[101] Mascan B., Marignol L. (2018). Aspirin in the Management of Patients with Prostate Cancer Undergoing Radiotherapy: Friend or Foe?. Anticancer Res..

[102] Hua H., Zhang H., Kong Q., Wang J., Jiang Y. (2019). Complex roles of the drug aspirin in cancer chemoprevention and therapy. Med. Res. Rev..

[103] Evans J.M., Donnelly L.A., Emslie-Smith A.M., Alessi D.R., Morris A.D. (2005). Metformin and reduced risk of cancer in diabetic patients. BMJ.

[104] Bowker S.L., Majumdar S.R., Veugelers P., Johnson J.A. (2006). Increased cancer-related mortality for patients with type 2 diabetes who use sulfonylureas or insulin. Diabetes Care.

[105] He X.X., Tu S.M., Lee M.H., Yeung S.C. (2011). Thiazolidinediones and metformin associated with improved survival of diabetic prostate cancer patients. Ann. Oncol..

[106] Margel D., Urbach D.R., Lipscombe L.L., Bell C.M., Kulkarni G., Austin P.C., Fleshner N. (2013). Metformin use and all-cause and prostate cancer-specific mortality among men with diabetes. J. Clin. Oncol..

[107] Wright J.L., Stanford J.L. (2009). Metformin use and prostate cancer in Caucasian men: Results from a population-based case-control study. Cancer Causes Control.

[108] Nobes J.P., Langley S.E., Klopper T., Russell-Jones D., Laing R.W. (2012). A prospective, randomized pilot study evaluating the effects of metformin and lifestyle intervention on patients with prostate cancer receiving androgen deprivation therapy. BJU Int..

[109] Stopsack K.H., Ziehr D.R., Rider J.R., Giovannucci E.L. (2016). Metformin and prostate cancer mortality: A meta-analysis. Cancer Causes Control.

[110] Xiao Y., Zheng L., Mei Z., Xu C., Liu C., Chu X., Hao B. (2017). The impact of metformin use on survival in prostate cancer: A systematic review and meta-analysis. Oncotarget.

[111] Richards K.A., Liou J.I., Cryns V.L., Downs T.M., Abel E.J., Jarrard D.F. (2018). Metformin use is associated with improved survival for patients with advanced prostate cancer on androgen deprivation therapy. J. Urol..

[112] He K., Hu H., Ye S., Wang H., Cui R., Yi L. (2019). The effect of metformin therapy on incidence and prognosis in prostate cancer: A systematic review and meta-analysis. Sci. Rep..

[113] Häggström C., Van Hemelrijck M., Zethelius B., Robinson D., Grundmark B., Holmberg L., Gudbjörnsdottir S., Garmo H., Stattin P. (2017). Prospective study of Type 2 diabetes mellitus, anti-diabetic drugs and risk of prostate cancer. Int. J. Cancer..

[114] Ghiasi B., Sarokhani D., Najafi F., Motedayen M., Dehkordi A.H. (2019). The Relationship between Prostate Cancer and Metformin Consumption: A Systematic Review and Meta-Analysis study. Curr. Pharm. Des..

[115] Owen M.R., Doran E., Halestrap A.P. (2000). Evidence that metformin exerts its anti-diabetic effects through inhibition of complex 1 of the mitochondrial respiratory chain. Biochem. J..

[116] Hardie D.G., Ross F.A., Hawley S.A. (2012). AMPK: A nutrient and energy sensor that maintains energy homeostasis. Nat. Rev. Mol. Cell Biol..

[117] Ben Sahra I., Le Marchand-Brustel Y., Tanti J.F., Bost F. (2010). Metformin in cancer therapy: A new perspective for an old antidiabetic drug?. Mol. Cancer Ther..

[118] Colquhoun A.J., Venier N.A., Vandersluis A.D., Besla R., Sugar L.M., Kiss A., Fleshner N.E., Pollak M., Klotz L.H., Venkateswaran V. (2012). Metformin enhances the antiproliferative and apoptotic effect of bicalutamide in prostate cancer. Prostate Cancer Prostatic Dis..

[119] Joshua A.M., Zannella V.E., Downes M.R., Bowes B., Hersey K., Koritzinsky M., Schwab M., Hofmann U., Evans A., van der Kwast T. (2014). A pilot ‘window of opportunity’ neoadjuvant study of metformin in localised prostate cancer. Prostate Cancer Prostatic Dis..

[120] Demir U., Koehler A., Schneider R., Schweiger S., Klocker H. (2014). Metformin anti-tumor effect via disruption of the MID1 translational regulator complex and AR downregulation in prostate cancer cells. BMC Cancer.

[121] Wang Y., Liu G., Tong D., Parmar H., Hasenmayer D., Yuan W., Zhang D., Jiang J. (2015). Metformin represses androgen-dependent and androgen-independent prostate cancers by targeting androgen receptor. Prostate.

[122] Iliopoulos D., Hirsch H.A., Struhl K. (2011). Metformin decreases the dose of chemotherapy for prolonging tumor remission in mouse xenografts involving multiple cancer cell types. Cancer Res..

[123] Zingales V., Distefano A., Raffaele M., Zanghi A., Barbagallo I., Vanella L. (2017). Metformin: A Bridge between Diabetes and Prostate Cancer. Front. Oncol..

[124] Zaidi S., Gandhi J., Joshi G., Smith N.L., Khan S.A. (2019). The anticancer potential of metformin on prostate cancer. Prostate Cancer Prostatic Dis..

[125] Sarmento-Cabral A., L-López F., Gahete M.D., Castaño J.P., Luque R.M. (2017). Metformin reduces prostate tumor growth, in a diet-dependent manner, by modulating multiple signaling pathways. Mol. Cancer Res..

[126] Whitburn J., Edwards C.M., Sooriakumaran P. (2017). Metformin and Prostate Cancer: A New Role for an Old Drug. Curr. Urol. Rep..

[127] Chan K.K., Oza A.M., Siu L.L. (2003). The statins as anticancer agents. Clin. Cancer Res..

[128] Sassano A., Platanias L.C. (2008). Statins in tumor suppression. Cancer Lett..

[129] Graaf M.R., Beiderbeck A.B., Egberts A.C., Richel D.J., Guchelaar H.J. (2004). The risk of cancer in users of statins. J. Clin. Oncol..

[130] Kaye J.A., Jick H. (2004). Statin use and cancer risk in the General Practice Research Database. Br. J. Cancer..

[131] Platz E.A., Tangen C.M., Goodman P.J., Till C., Parnes H.L., Figg W.D., Albanes D., Neuhouser M.L., Klein E.A., Lucia M.S. (2014). Statin drug use is not associated with prostate cancer risk in men who are regularly screened. J. Urol..

[132] Tan P., Zhang C., Wei S.Y., Tang Z., Gao L., Yang L., Wei Q. (2017). Effect of statins type on incident prostate cancer risk: A meta-analysis and systematic review. Asian J. Androl..

[133] Shannon J., Tewoderos S., Garzotto M., Beer T.M., Derenick R., Palma A., Farris P.E. (2005). Statins and prostate cancer risk: A case-control study. Am. J. Epidemiol..

[134] Tan N., Klein E.A., Li J., Moussa A.S., Jones J.S. (2011). Statin use and risk of prostate cancer in a population of men who underwent biopsy. J. Urol..

[135] Bansal D., Undela K., D’Cruz S., Schifano F. (2012). Statin use and risk of prostate cancer: A meta-analysis of observational studies. PLoS ONE.

[136] Jespersen C.G., Nørgaard M., Friis S., Skriver C., Borre M. (2014). Statin use and risk of prostate cancer: A Danish population-based case-control study, 1997-2010. Cancer Epidemiol..

[137] Babcook M.A., Joshi A., Montellano J.A., Shankar E., Gupta S. (2016). Statin Use in Prostate Cancer: An Update. Nutr. Metab. Insights.

[138] Nielsen S.F., Nordestgaard B.G., Bojesen S.E. (2012). Statin use and reduced cancer-related mortality. N. Engl. J. Med..

[139] Yu O., Eberg M., Benayoun S., Aprikian A., Batist G., Suissa S., Azoulay L. (2014). Use of statins and the risk of death in patients with prostate cancer. J. Clin. Oncol..

[140] Tan P., Wei S., Yang L., Tang Z., Cao D., Liu L., Lei J., Fan Y., Gao L., Wei Q. (2016). The effect of statins on prostate cancer recurrence and mortality after definitive therapy: A systematic review and meta-analysis. Sci. Rep..

[141] Raval A.D., Thakker D., Negi H., Vyas A., Kaur H., Salkini M.W. (2016). Association between statins and clinical outcomes among men with prostate cancer: A systematic review and meta-analysis. Prostate Cancer Prostatic Dis..

[142] Marcella S.W., David A., Ohman-Strickland P.A., Carson J., Rhoads G.G. (2012). Statin use and fatal prostate cancer: A matched case-control study. Cancer.

[143] Pon D., Abe A., Gupta E.K. (2015). A review of statin use and prostate cancer. Curr. Atheroscler. Rep..

[144] Papadopoulos G., Delakas D., Nakopoulou L., Kassimatis T. (2011). Statins and prostate cancer: Molecular and clinical aspects. Eur. J. Cancer.

[145] Alfaqih M.A., Allott E.H., Hamilton R.J., Freeman M.R., Freedland S.J. (2017). The current evidence on statin use and prostate cancer prevention: Are we there yet?. Nat. Rev. Urol..

[146] Simons K., Ikonen E. (1997). Functional rafts in cell membranes. Nature.

[147] Freeman M.R., Cinar B., Lu M.L. (2005). Membrane rafts as potential sites of nongenomic hormonal signaling in prostate cancer. Trends Endocrinol. Metab..

[148] Zhuang L., Lin J., Lu M.L., Solomon K.R., Freeman M.R. (2002). Cholesterol-rich lipid rafts mediate akt-regulated survival in prostate cancer cells. Cancer Res..

[149] Bañez L.L., Klink J.C., Jayachandran J., Lark A.L., Gerber L., Hamilton R.J., Masko E.M., Vollmer R.T., Freedland S.J. (2010). Association between statins and prostate tumor inflammatory infiltrate in men undergoing radical prostatectomy. Cancer Epidemiol. Biomarkers Prev..

[150] Allott E.H., Howard L.E., Vidal A.C., Moreira D.M., Castro-Santamaria R., Andriole G.L., Freedland S.J. (2017). Statin use, serum lipids, and prostate inflammation in men with a negative prostate biopsy: Results from the REDUCE Trial. Cancer Prev. Res..

[151] Murtola T.J., Syvälä H., Tolonen T., Helminen M., Riikonen J., Koskimäki J., Pakarainen T., Kaipia A., Isotalo T., Kujala P. (2018). Atorvastatin versus placebo for prostate cancer before radical prostatectomy-a randomized, double-blind, placebo-controlled clinical trial. Eur. Urol..

[152] Ridker P.M. (2016). From C-Reactive Protein to Interleukin-6 to Interleukin-1: Moving Upstream To Identify Novel Targets for Atheroprotection. Circ. Res..

[153] Sugiyama M., Ohashi M., Takase H., Sato K., Ueda R., Dohi Y. (2005). Effects of atorvastatin on inflammation and oxidative stress. Heart Vessels..

[154] Mausner-Fainberg K., Luboshits G., Mor A., Maysel-Auslender S., Rubinstein A., Keren G., George J. (2008). The effect of HMG-CoA reductase inhibitors on naturally occurring CD4+CD25+ T cells. Atherosclerosis.

[155] Van Die M.D., Bone K.M., Williams S.G., Pirotta M.V. (2014). Soy and soy isoflavones in prostate cancer: A systematic review and meta-analysis of randomized controlled trials. BJU Int..

[156] Bosland M.C., Kato I., Zeleniuch-Jacquotte A., Schmoll J., Enk Rueter E., Melamed J., Kong M.X., Macias V., Kajdacsy-Balla A., Lumey L.H. (2013). Effect of soy protein isolate supplementation on biochemical recurrence of prostate cancer after radical prostatectomy: A randomized trial. JAMA.

[157] Rivero J.R., Thompson I.M., Liss M.A., Kaushik D. (2018). Chemoprevention in Prostate Cancer: Current Perspective and Future Directions. Cold Spring Harb. Perspect Med..

[158] Lin P.H., Aronson W., Freedland S.J. (2019). An update of research evidence on nutrition and prostate cancer. Urol. Oncol..

